# Supplementary figures and images for: 5 Years of bipolar disorder conversations on Reddit: Methods, key topics and future directions
Source: PLoS One. 2026 Mar 6;21(3):e0338622. doi: 10.1371/journal.pone.0338622 (PMC12965559; doi:10.1371/journal.pone.0338622)

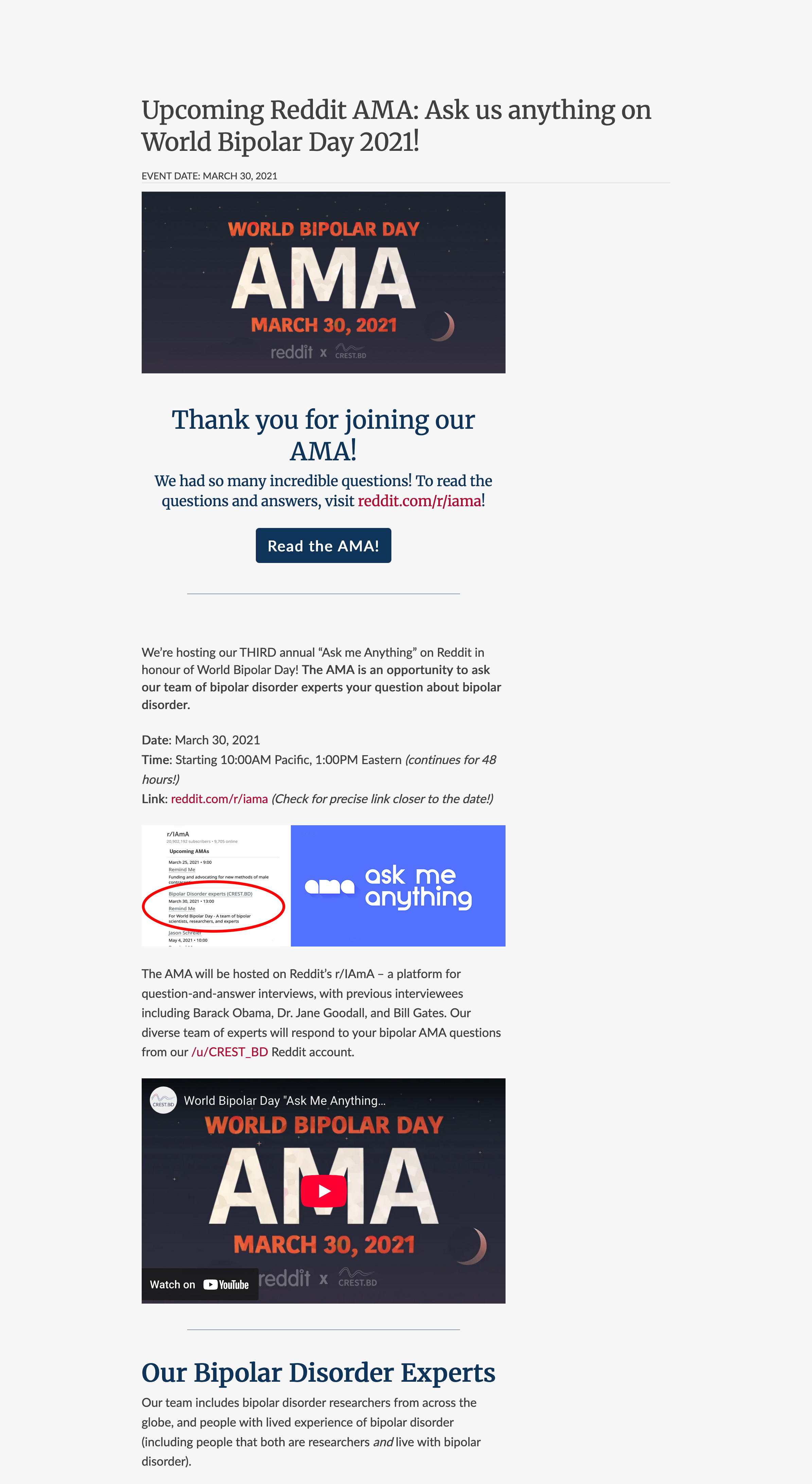

Supplement: S1 File — (JPG) [file pone.0338622.s001.jpg]

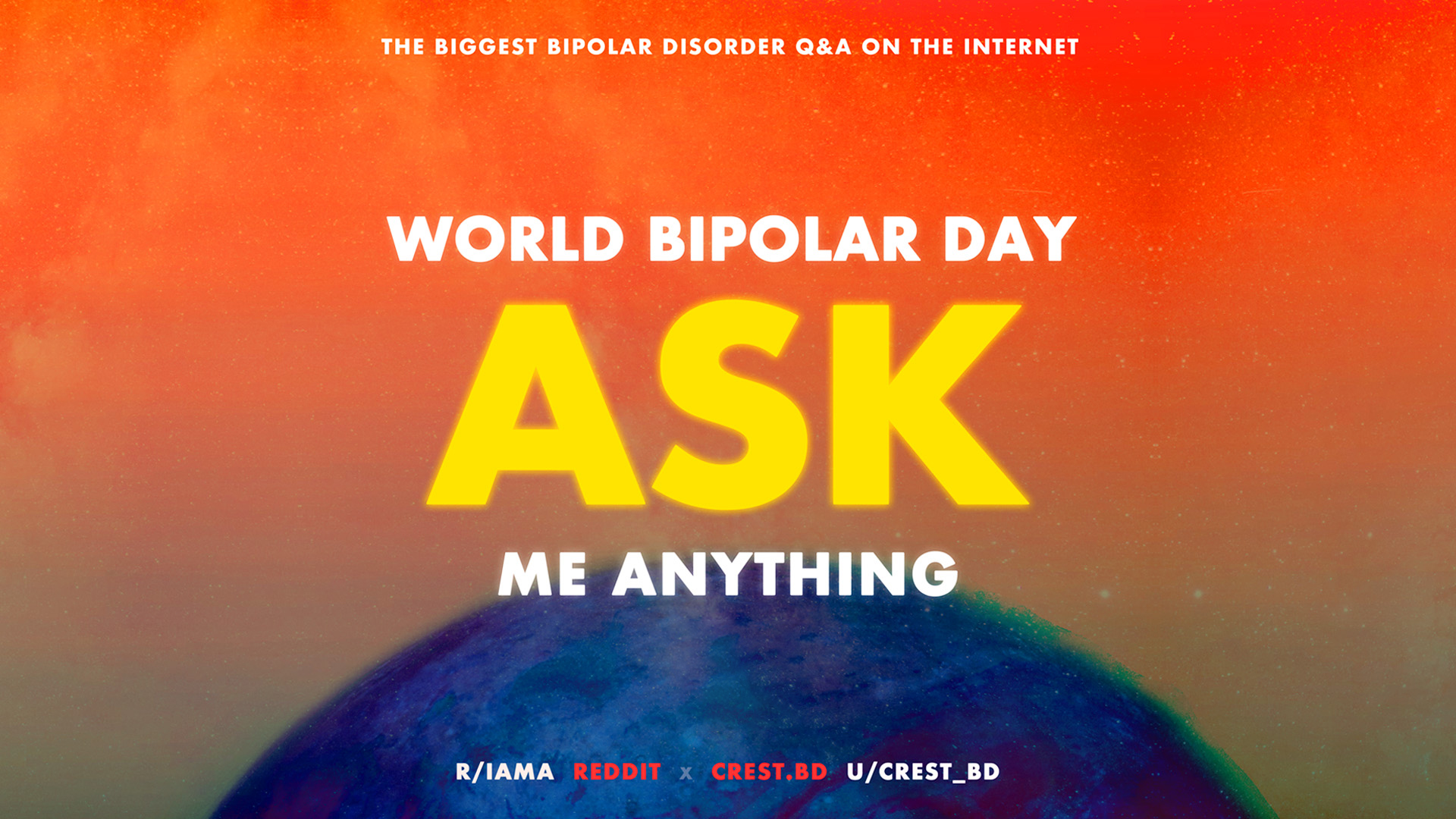

Supplement: S2 File — (JPG) [file pone.0338622.s002.jpg]
