# Supplementary material for: 5 Years of bipolar disorder conversations on Reddit: Methods, key topics and future directions
Source: PLoS One. 2026 Mar 6;21(3):e0338622. doi: 10.1371/journal.pone.0338622 (PMC12965559; doi:10.1371/journal.pone.0338622)
Supplement: Attachment — Submitted filename: Response to Reviewers [PONE-D-25-19143].docx [file pone.0338622.s004.docx]

Thank you for reviewing our manuscript. This rebuttal letter contains responses to each point.

**1. Please ensure that your manuscript meets PLOS ONE's style requirements, including those for file naming. The PLOS ONE style templates can be found at**

[**https://journals.plos.org/plosone/s/file?id=wjVg/PLOSOne_formatting_sample_main_body.pdf**](https://journals.plos.org/plosone/s/file?id=wjVg/PLOSOne_formatting_sample_main_body.pdf) **and**

[**https://journals.plos.org/plosone/s/file?id=ba62/PLOSOne_formatting_sample_title_authors_affiliations.pdf**](https://track.editorialmanager.com/CL0/https:%2F%2Fjournals.plos.org%2Fplosone%2Fs%2Ffile%3Fid=ba62%2FPLOSOne_formatting_sample_title_authors_affiliations.pdf/1/010f019763ac6b0a-889a617d-1d2c-41a1-8c22-8807b35aa40c-000000/pE1xMa-1vhiCuFdZd87sPJwcMFHfLpPJYFyHvFAnnDM=215)

Our manuscript has been styled to meet the PLOS ONE style requirements.

**2. Thank you for stating the following financial disclosure: [CREST.BD education outreach activities are support by Otsuka-Lundbeck unrestricted grants.]**

**Please state what role the funders took in the study. If the funders had no role, please state: ""The funders had no role in study design, data collection and analysis, decision to publish, or preparation of the manuscript.""**

**If this statement is not correct you must amend it as needed.**

**Please include this amended Role of Funder statement in your cover letter; we will change the online submission form on your behalf.**

The funders had no role in the study, we now state in the amended cover letter that "The funders had no role in study design, data collection and analysis, decision to publish, or preparation of the manuscript.

**3. One of the noted authors is a group. [The Collaborative RESearch Team to study psychosocial issues in Bipolar Disorder (CREST.BD)]. In addition to naming the author group, please list the individual authors and affiliations within this group in the acknowledgments section of your manuscript. Please also indicate clearly a lead author for this group along with a contact email address.**

This has now been added to the acknowledgments section of the manuscript.

**4. Please include your full ethics statement in the ‘Methods’ section of your manuscript file. In your statement, please include the full name of the IRB or ethics committee who approved or waived your study, as well as whether or not you obtained informed written or verbal consent. If consent was waived for your study, please include this information in your statement as well.**

We thank the Editor for this comment. We took a considered approach to the ethics of this paper. As we explain in lines 221-226, for data of this type (large volumes of anonymous online data), implementing typical consent procedures was not determined to be feasible. Instead, as we detail in the paper, we have taken a considerate approach to using this data in a way that respects confidentiality and privacy, e.g. removing all usernames and paraphrasing all quotes. We hope that this is satisfying to the Editor and are happy to discuss the matter further if required.

**5. Please remove all personal information, ensure that the data shared are in accordance with participant consent, and re-upload a fully anonymized data set.**

**Note: spreadsheet columns with personal information must be removed and not hidden as all hidden columns will appear in the published file.**

**Additional guidance on preparing raw data for publication can be found in our Data Policy (https://journals.plos.org/plosone/s/data-availability#loc-human-research-participant-data-and-other-sensitive-data) and in the following article:** [**http://www.bmj.com/content/340/bmj.c181.long**](http://track.editorialmanager.com/CL0/http:%2F%2Fwww.bmj.com%2Fcontent%2F340%2Fbmj.c181.long/1/010f019763ac6b0a-889a617d-1d2c-41a1-8c22-8807b35aa40c-000000/NOi73-OgH6XI78LjsCYfZX8g2khjQVQpzjwQGmhqzDM=215)**.**

Personal information is not included in the data set contained in the manuscript.

**6. We note that [Supplementary File 2 - Blog with Proof Photos] includes an image of a participant in the study].**

**As per the PLOS ONE policy (http://journals.plos.org/plosone/s/submission-guidelines#loc-human-subjects-research) on papers that include identifying, or potentially identifying, information, the individual(s) or parent(s)/guardian(s) must be informed of the terms of the PLOS open-access (CC-BY) license and provide specific permission for publication of these details under the terms of this license. Please download the Consent Form for Publication in a PLOS Journal (http://journals.plos.org/plosone/s/file?id=8ce6/plos-consent-form-english.pdf). The signed consent form should not be submitted with the manuscript, but should be securely filed in the individual's case notes. Please amend the methods section and ethics statement of the manuscript to explicitly state that the patient/participant has provided consent for publication: “The individual in this manuscript has given written informed consent (as outlined in PLOS consent form) to publish these case details”.**

**If you are unable to obtain consent from the subject of the photograph, you will need to remove the figure and any other textual identifying information or case descriptions for this individual.**

All photos and identifiable information, including images of panelists have now been removed from [Supplementary File 2 - Blog with Proof Photos]. This has now been replaced with a new attachment [S2 Supplementary Information. Promotional Blog].

**7. Please include captions for your Supporting Information files at the end of your manuscript, and update any in-text citations to match accordingly. Please see our Supporting Information guidelines for more information:** [**http://journals.plos.org/plosone/s/supporting-information**](http://track.editorialmanager.com/CL0/http:%2F%2Fjournals.plos.org%2Fplosone%2Fs%2Fsupporting-information/1/010f019763ac6b0a-889a617d-1d2c-41a1-8c22-8807b35aa40c-000000/EPyHQz-QEFkwREErbEzulRVX48GZeDylco6F9hXSw_g=215)**.**

Supporting Information file names have now been updated according to the guidelines.

**8. Please review your reference list to ensure that it is complete and correct. If you have cited papers that have been retracted, please include the rationale for doing so in the manuscript text, or remove these references and replace them with relevant current references. Any changes to the reference list should be mentioned in the rebuttal letter that accompanies your revised manuscript. If you need to cite a retracted article, indicate the article’s retracted status in the References list and also include a citation and full reference for the retraction notice.**

The reference list is complete and correct. No cited papers have been retracted.
